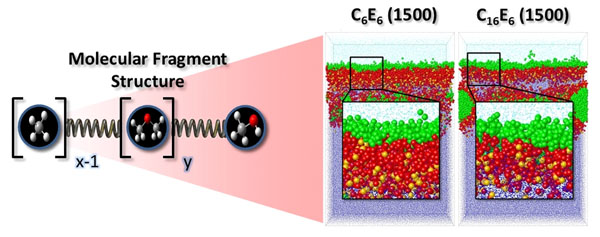# Molecular fragment dynamics study on the water-air interface behavior of non-ionic polyoxyethylene alkyl ether surfactants

**DOI:** 10.1186/1758-2946-6-S1-P9

**Published:** 2014-03-11

**Authors:** Andreas Truszkowski, Annamaria Fiethen, Hubert Kuhn, Thomas Wiebringhaus, Achim Zielesny, Matthias Epple

**Affiliations:** 1 Inorganic Chemistry and Center for Nanointegration, University of Duisburg-Essen, Essen, 45141, Germany; 2 CAM-D Technologies, Essen, 45117, Germany; 3 Institute for Bioinformatics and Chemoinformatics, Westphalian University of Applied Sciences, Recklinghausen, 45665, Germany

## 

Molecular Fragment Dynamics (MFD) is a mesoscopic simulation technique based on Dissipative Particle Dynamics (DPD). Whereas DPD beads in general may not necessarily be identified with chemical compounds at all the MFD variant uses specific molecules or molecular fragments as its basic *coarse-grained* interacting entities (rather than the *fine-grained* atom types of Molecular Mechanics). MFD can be used to study formulations of drugs and active agents in oil, water and emulsions.

MFD simulations of the nonionic polyoxyethylene alkyl ether surfactants C_6_E_6_, C_10_E_6_, C_12_E_6_ and C_16_E_6_ at the water-air interface are performed to study their nanoscale structures and surface properties. The simulations of the self-aggregation of the polyoxyethylene alkyl ether surfactants lead to equilibrium nanoscale structures and computationally determined surface tensions which are in agreement with experimental data for different surfactant concentrations [1].

**Figure 1:**